# Bone Components Downregulate Expression of Toll-Like Receptor 4 on the Surface of Human Monocytic U937 Cells: A Cell Model for Postfracture Immune Dysfunction

**DOI:** 10.1155/2015/896576

**Published:** 2015-07-26

**Authors:** Jui-An Lin, Feng-Yen Lin, Ta-Liang Chen

**Affiliations:** ^1^Graduate Institute of Clinical Medicine, College of Medicine, Taipei Medical University, Taipei 11031, Taiwan; ^2^Department of Anesthesiology, School of Medicine, College of Medicine, Taipei Medical University, Taipei 11031, Taiwan; ^3^Department of Anesthesiology, Taipei Medical University Hospital, Taipei 11031, Taiwan; ^4^Department of Internal Medicine, School of Medicine, College of Medicine, Taipei Medical University, Taipei 11031, Taiwan

## Abstract

To mimic the immune status of monocyte in the localized fracture region, toll-like receptor 4 (TLR4) surface expression in human monocytic U937 cells was used as the main target to assess immune dysfunction following bone component exposure. We first identified the effects of bone components (including the marrow content) on TLR4 surface expression and then examined the mechanisms underlying the changes. The level of microRNA-146a expression, an indicator of endotoxin tolerance, was also assayed. Bone component exposure downregulated TLR4 surface expression at 24 h by flow cytometry analysis, compatible with the result obtained from the membranous portion of TLR4 by western blot analysis. The cytoplasmic portion of TLR4 paradoxically increased after bone component exposure. Impaired TLR4 trafficking from the cytoplasm to the membrane was related to gp96 downregulation, as observed by western blot analysis, and this was further evidenced by gp96-TLR4 colocalization under confocal microscopy. TaqMan analysis revealed that the expression of microRNA-146a was also upregulated. This cell model demonstrated that bone component exposure downregulated TLR4 surface expression in a gp96-related manner in human monocytic U937 cells, an indicator of immunosuppression at 24 h. Immune dysfunction was further evidenced by upregulation of microRNA-146a expression at the same time point.

## 1. Introduction

Based on projections of economic and social development, it is estimated that, by 2030, trauma will account for one of the major disease burdens worldwide, possibly even ahead of ischemic heart disease [1]. Trauma is the leading cause of death among people aged 15 through 44 years [2, 3]. Sepsis after trauma increases the rate of organ failure and in-hospital mortality. Despite advances in trauma care and research, the incidence of posttraumatic sepsis still remained as high as 10% over the last decade [4]. The general lack of effective therapeutic options for trauma patients is mainly due to the complexity of interacting inflammatory and physiological components at multiple levels [5, 6]. Therefore, focusing on the impact of individual components helps to evaluate trauma conditions in depth.

Approximately 90% of multiple trauma patients show long-bone fractures [7] and patients dying from sepsis after trauma frequently have orthopedic injuries [8]; thus, orthopedic trauma is a major specialty in the field of trauma research. Multiple superimposed insults contribute to a complicated postfracture immune alteration, including the fracture itself and related surgery/anesthesia; soft tissue trauma and accompanying ischemia reperfusion injury; and hemorrhage and ensuing blood transfusion [9]. Besides, trauma alone accounts for a substantial proportion of genetic changes and the addition of hemorrhagic shock only increases the magnitude of the expression with relatively few extra genes recruited [5]. Evidence shows that the fracture itself depresses both the innate and adaptive immune functions [10, 11]. As a result, a localized immune microenvironment is created [12] and the overspill of local trauma mediators may be responsible for the systemic alteration of trauma-specific cytokines [13]. An earlier study also demonstrated that the wound fluid might be the source of posttraumatic immunosuppression, which is related to mortality [14]. Monocytes predominate within the hematoma at the fracture site, and suppression of their antigen-presenting capacity is also found to be greater than that in the peripheral blood [12], indicating that monocytes might play a key role in posttraumatic immunosuppression. Furthermore, damage-associated molecular patterns, such as pathogen-recognition molecular patterns and alarmins, activate the innate immune response after trauma primarily through toll-like receptors (TLR) [15]. More than any one of the other TLR family members, TLR4 sits at the interface of microbial and sterile (such as trauma) inflammation [16] and variation within TLR4 gene is associated with severity of posttraumatic sepsis [17]. As described above, TLR4 expression of monocytes at the localized fracture site might direct the immune status in trauma patients.

A pseudofracture model developed by bone component injection into thighs has been used to help recovery of rodents from trauma for longer-term studies [18–20]. However, no specific cell model is available for in-depth investigations of related mechanisms and possible gene manipulation. We aimed to use TLR4 surface expression on bone component-treated monocytes as the target to mimic the localized postfracture immune condition.

## 2. Materials and Methods

### 2.1. Extraction of Bone Components

The procedure for preparing bone components was modified from the pseudofracture model [18–20]. C57BL/6 male mice were sacrificed and 2 femurs and 2 tibias from the lower extremities were harvested under sterile conditions. The bones were crushed using a triturator and suspended in 2 mL of PBS. This suspension was then homogenized and also flushed through a 70 *μ*m cell strainer (BD Biosciences, San Jose, CA, USA). The concentration of bone components was assayed using the Bradford Protein Assay Kit (Bio-Rad, Hercules, CA, USA) and stored at −80°C.

### 2.2. Cell Culture

U937 cells (human monocytic cell line) were obtained from the American Type Culture Collection (ATCC, Manassas, VA, USA) and grown in RPMI 1640 medium with 4.5 g/L glucose, 10 mmol/L HEPES, and 1.0 mmol/L sodium pyruvate, supplemented with 10% fetal bovine serum, 2 mM l-glutamine, and 1% antibiotic-antimycotic mixture. Cell density was maintained between 5 × 10^4^ and 8 × 10^5^ viable cells/mL, and the medium was replaced every 2 or 3 days.

### 2.3. Flow Cytometry Analysis

To examine membrane TLR4 expression on U937 cells, 1 × 10^5^ cells were incubated with a phycoerythrin-conjugated mouse anti-human TLR4 antibody (BioLegend, San Diego, CA, USA). After the cells were washed with staining buffer (PBS containing 0.1% bovine serum albumin and 0.1% sodium azide), TLR4 expression was analyzed on a fluorescence-activated cell sorting Calibur flow cytometer (Becton Dickinson, San Jose, CA, USA).

### 2.4. Quantitative Real-Time Polymerase Chain Reaction

Total RNA was isolated using the TRIzol Reagent (Invitrogen, Carlsbad, CA, USA), and cDNA was synthesized according to the manufacturer's instructions. The cross-point values were calculated in the quantitative real-time polymerase chain reaction (qPCR) to detect the presence of TLR4 mRNA against glyceraldehyde-3-phosphate dehydrogenase (GAPDH) as an internal control. The PCR primers used for the amplification of TLR4 and GAPDH were as in Table 1.

### 2.5. Actinomycin D Chase Experiment

Actinomycin D (10 *μ*g/mL) was added to the cells for 30 min following treatment under various experimental conditions. Total RNA was extracted at 30–360 min time points after the addition of actinomycin D, and qPCR was performed.

### 2.6. Western Blot Analysis

Total cell lysates were extracted from U937 cells. Proteins were separated by SDS-PAGE and transferred to a PVDF membrane. The membranes were probed with mouse anti-TLR4 (Abcam, Cambridge, MA, USA), mouse anti-*β*-actin (Labvision/NeoMarkers, Kalamazoo, MI, USA), goat anti-G*α*s (Santa Cruz, Dallas, TX, USA), mouse anti-protein associated with toll-like receptor 4 (PRAT4A) (Abcam), or chicken antiglycoprotein 96 (gp96) (GeneTex, Irvine, CA, USA) antibodies. The proteins were visualized with an enhanced chemiluminescence detection kit (Amersham Biosciences, Pittsburgh, PA, USA). Mouse anti-*β*-actin (Labvision/NeoMarkers) antibody was used as a loading control. Membrane and cytosolic fractions were separated according to the protocols described previously [21] with the extract lysed in the membrane protein lysis buffer (20 mM Tris-HCl, pH 8.0, 137 mM NaCl, 10% glycerol, 1% NP-40, 2 mM EDTA, and 1 mM phenylmethylsulfonyl fluoride (PMSF)). For the membrane fraction, rabbit anti-G*α*s (Abcam) antibody was used as the internal control.

### 2.7. Immunofluorescent Staining

Cells were fixed with 4% paraformaldehyde and quickly plated onto coverslips using a Shandon CytoSpin III Cytocentrifuge (GMI, Ramsey, MN, USA). Cell membranes were fenestrated with 0.4% Triton X-100-PBS, and nonspecific binding sites were blocked with 2% BSA-PBS-Tween 20 (0.1% v/v). Samples were stained with mouse anti-TLR4 (Abcam), mouse anti-PRAT4A (Abcam), or chicken anti-gp96 (GeneTex) antibodies and were incubated with Alexa-conjugated secondary antibodies. 4′,6-Diamidino-2-phenylindole stain was used to identify the cell nuclei. All slides were examined by confocal microscopy (LSM510, ZEISS Inc., Germany).

### 2.8. MicroRNA Expression Analysis

The mirVana miRNA Isolation Kit (Applied Biosystems, Grand Island, NY, USA) was used to extract the small RNA; the TaqMan microRNA Reverse Transcription Kit and TaqMan MicroRNA Assay Kit (Applied Biosystems) were used to analyze the expression of microRNA-146a (miRNA-146a).

### 2.9. Statistical Analysis

The values are expressed as the mean ± SEM. Data were analyzed using Student's *t*-test. Probability values (*P*) < 0.05 were considered to be significant.

## 3. Results

**Figure 1:**
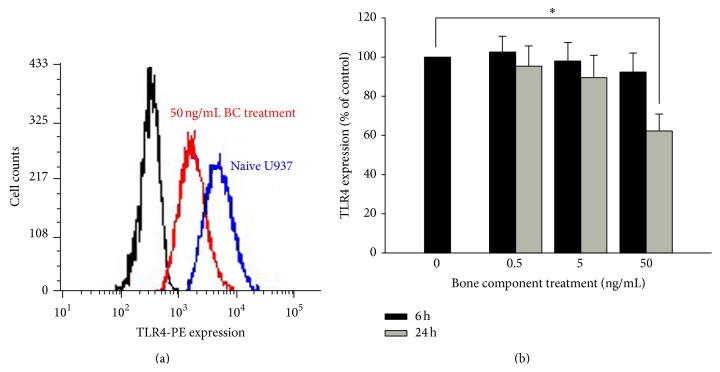
Downregulation of TLR4 surface expression by bone component (BC) exposure in U937 cells. (a) U937 cells were treated with (red graph) or without (blue graph) 50 ng/mL BC for 24 h. The TLR4 membranous portion was stained with a phycoerythrin-conjugated anti-TLR4 antibody and analyzed using flow cytometry. Negative control analyses were performed in the absence of the specific phycoerythrin-conjugated anti-TLR4 antibody (black graph). (b) U937 cells were treated with 0.5–50 ng/mL BC for 6 or 24 hrs. TLR4 levels in U937 cells were examined using flow cytometry, and the results are presented as percentages of the control. Data are presented as mean ± SEM (*n* = 3); ^∗^ *P* < 0.05 compared with the control group.

To test the effect of bone component treatment on monocytic TLR4 expression, we first examined the amount of functional TLR4, that is, cell surface expression. As shown in Figure 1, bone components did not alter TLR4 surface expression until 24 h exposure, and only the higher concentration of the bone component (50 ng/mL) induced downregulation of TLR4 expression.

**Figure 2:**
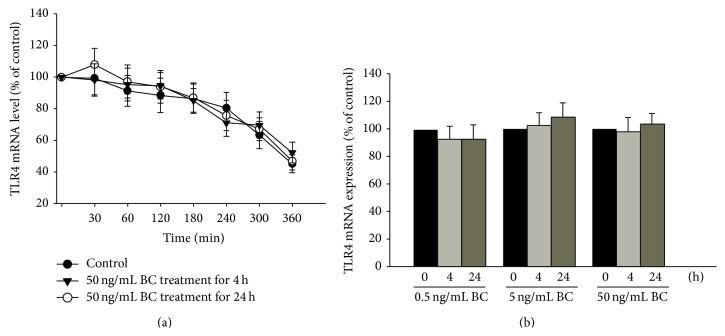
Regulation of TLR4 expression by bone component (BC) exposure in U937 cells not mediated by transcriptional and posttranscriptional modifications. (a) Actinomycin D chase experiment to evaluate the stability of TLR4 mRNA in 50 ng/mL BC-treated U937 cells. (b) U937 cells were treated with 0.5–50 ng/mL BC for the indicated time (4 or 24 h). TLR4 mRNA levels were analyzed by quantitative real-time PCR after normalization to GAPDH mRNA. Data are presented as mean ± SEM (*n* = 3).

**Figure 3:**
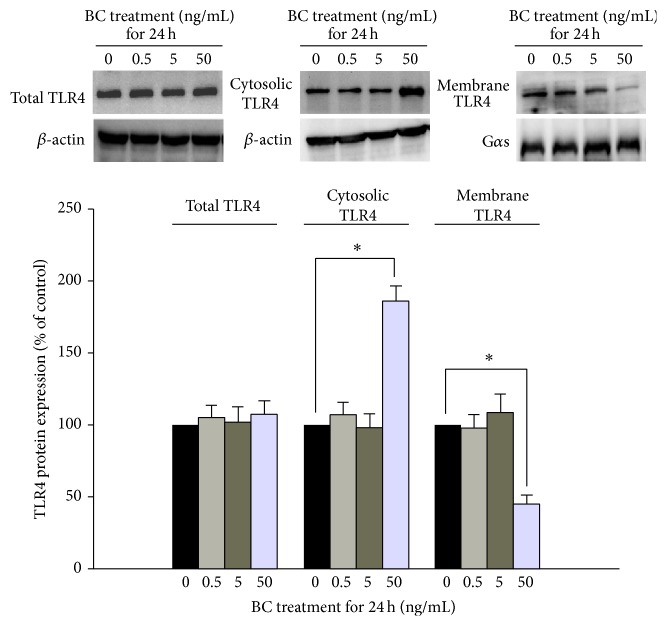
Alteration of subcellular distribution of TLR4 protein by bone component (BC) exposure in U937 cells. U937 cells were treated with 0.5–50 ng/mL BC for 24 hr. Total protein, cytoplasmic portion, and membranous portion of TLR4 in U937 cells were detected using western blot analysis. The *β*-actin and G*α*s were used as an internal control. The bar diagram represents quantification of TLR4 expression in U937 cells. Data represent the results of 3 independent experiments (mean ± SEM; ^∗^ *P* < 0.05 between groups).

**Figure 4:**
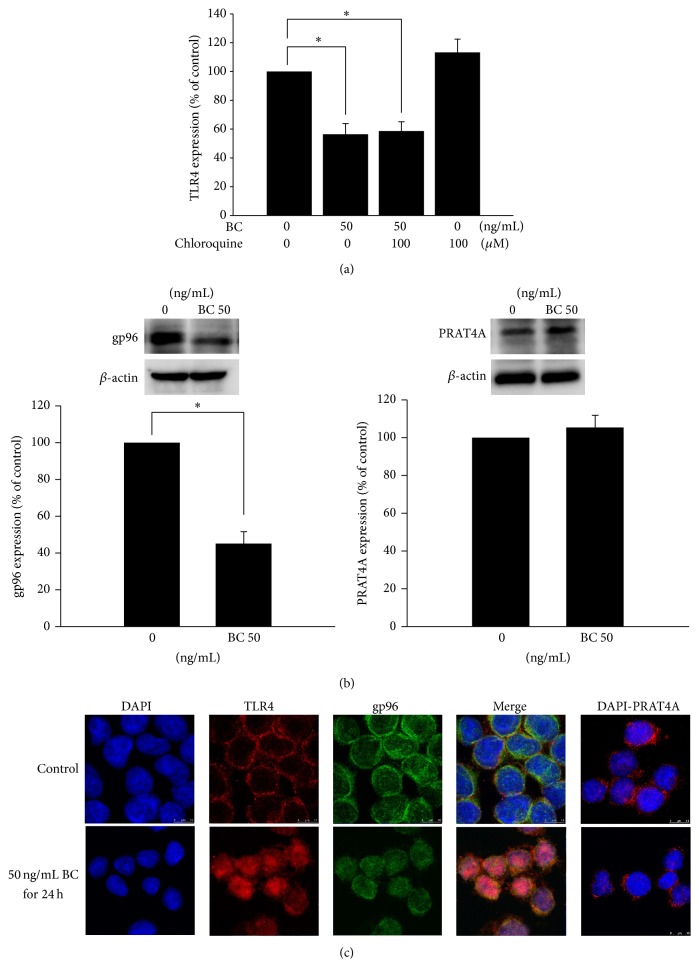
Involvement of glycoprotein 96 (gp96) but not protein associated with toll-like receptor 4 (PRAT4A) in the downregulation of TLR4 surface expression in U937 cells after bone component (BC) exposure. (a) U937 cells were pretreated with 100 *μ*M chloroquine for 2 h before 50 ng/mL BC treatment for 24 h. Membranous portion of TLR4 in U937 cells was examined using flow cytometry, and results are presented as percentages of the control. Data are presented as mean ± SEM (*n* = 3); ^∗^ *P* < 0.05 compared with the control group. (b) U937 cells were treated with 50 ng/mL BC for 24 h. Cellular gp96 and PRAT4A in U937 cells were detected using western blot analysis. *β*-actin was used as an internal control. Data represent the results of 3 independent experiments (mean ± SEM; ^∗^ *P* < 0.05 was considered significant). (c) U937 cells were stimulated with 50 ng/mL BC for 24 h. The gp96, PRAT4A, and TLR4 proteins were identified with specific antibodies by immunofluorescence. 4′,6-Diamidino-2-phenylindole was used to characterize the nucleus. The slides were observed by confocal microscopy.

Downregulation of TLR4 expression may be either globally or regionally regulated. TLR4 mRNA (transcriptional level) and mRNA stability (posttranscriptional level) were not altered during the 24 h bone component exposure (Figure 2). Furthermore, total TLR4 protein expression was not affected by bone component exposure (Figure 3), thus validating the fact that regional changes in TLR4 could account for the downregulation of its surface expression. The subcellular distribution was further accessed by TLR4 protein amount. The expression of the membranous portion of the TLR4 protein decreased in cells treated with a moderately high concentration (50 ng/mL) of bone components, which was consistent with the flow cytometry result. However, the expression of the cytoplasmic portion of the TLR4 protein increased with bone component exposure as compared to the control (Figure 3), suggesting that TLR4 protein accumulation within the cytoplasm is a possible cause of decreased membranous expression. According to recent comprehensive reviews regarding the mechanisms of TLR4 localization [22, 23], trafficking from the endoplasmic reticulum to the membrane and lysosome-mediated TLR4 degradation are 2 major ways of controlling TLR4 surface expression.  Figure 4(a) showed that pretreatment with high-dose lysosomal inhibitor, chloroquine, did not alter the downregulation of membranous TLR4 expression after 50 ng/mL bone component exposure, thus ruling out a protein-degradation pathway and favoring a protein-accumulation mechanism (in combination with Figure 3) to explain the bone component-mediated TLR4 downregulation. Chaperones currently known to regulate TLR4 localization and surface expression, for example, gp96 and PRAT4A [23], were examined to determine if their expressions changed with bone component exposure. We found that, unlike PRAT4A expression, gp96 expression decreased at a moderately high concentration (50 ng/mL) of bone components (Figure 4(b)). Localization of TLR4, gp96, and PRAT4A was also detected by immunofluorescence and observed by confocal microscopy. Impaired TLR4 trafficking from the cytoplasm to the membrane and colocalization of TLR4 with cytoplasmic gp96 were found after bone component exposure (Figure 4(c)).

**Figure 5:**
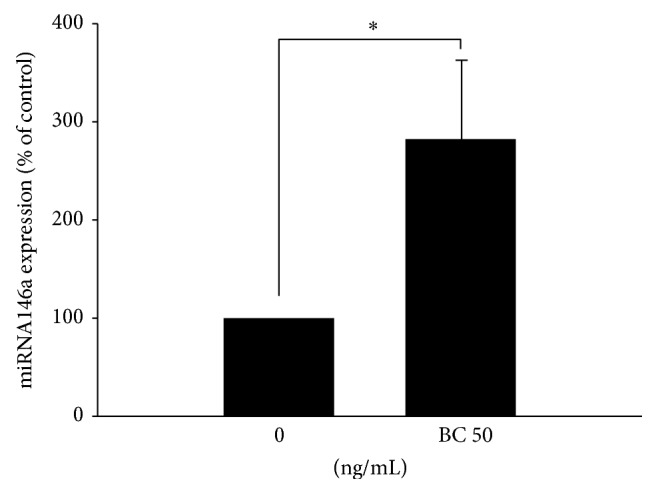
Upregulation of miRNA-146a expression in U937 cells after bone component (BC) exposure. U937 cells were treated with 50 ng/mL BC for 24 h. The levels of miRNA-146a in U937 cells were analyzed by quantitative real-time PCR after normalization to U6 small nuclear RNA. Data are presented as means ± SEM (*n* = 4); ^∗^ *P* < 0.05 compared with the control group.

After elucidating the mechanism underlying the downregulation of TLR4 surface expression, we further determined if miR-146a, the major contributing factor to endotoxin tolerance [24], was also involved in the immunosuppressive consequence in this postfracture cell model. After exposure to a moderately high concentration (50 ng/mL) of bone components, upregulation of miR-146a expression was found (Figure 5) to support the existence of endotoxin tolerance at this time point in the cell model.

## 4. Discussion

We successfully developed a cell model assessing the impact of bone components on the immune status of monocytes. TLR4 surface expression on human monocytes was downregulated after exposure to bone components for 24 h. After excluding the possibilities of altered transcription, posttranscriptional modification, and degradation, we found that membranous TLR4 downregulation resulted from impaired translocation from the cytoplasm to the membrane in a gp96-related manner. A negative regulator of TLR4 signaling, miR-146a, was also found to be upregulated following exposure to bone components.

As the maxim states, “to every action there is always opposed an equal reaction.” A complicated manifestation of this general physics principle is found in the human body, which can mount an overreacted, an underreacted, or a balanced reaction to trauma [25]. Compensatory anti-inflammatory response syndrome is postulated to occur either in a 2-wave process after or concomitantly with systemic inflammatory response syndrome [26]. As the compensatory anti-inflammatory response syndrome predominates, trauma results in immunosuppression and increases the susceptibility to sepsis [25].

A genome-wide expression analysis regarding trauma-induced multiorgan dysfunction syndrome reveals that molecular interactions exist between monocytes and T cells, including increased expression of inhibitory costimulation receptor/ligand combinations and decreased expression of stimulatory receptor/ligand combinations between monocytes and T cells [27]. Among the various interactions, prolonged monocyte dysfunction has been proposed to initiate a cascade of septic complications [28]. A retrospective analysis carried out in trauma patients with human immunodeficiency virus infection shows that posttraumatic bacterial infection correlates only with the injury severity, not the progression severity of human immunodeficiency virus [29]. This indicates that mechanisms other than those involving CD4^+^ T lymphocytes, such as the monocyte being the research target in our model, may be the leading mechanisms in posttraumatic immunosuppression. As for the choice of functional indicators in the trauma monocyte model, human leukocyte antigen- (HLA-) DR expression used to be one of them to examine the decrease of antigen-presenting capacity in the trauma setting [28]. However, conflicting results exist regarding the correlation between monocyte HLA-DR expression and trauma outcome, and* ex vivo* lipopolysaccharide- (LPS-) induced cytokine release seems to be an earlier and better predictor of patient survival than HLA-DR expression in “traumatized” monocytes [30]. Among the receptors necessary for LPS signaling of patient's monocytes, that is, TLR4 and TLR2, only TLR4 expression is selectively impaired by trauma [9]. TLR4 is also the key to sterile inflammation and remote organ dysfunction (such as postfracture acute lung injury) [19] and both immunosuppression and hyperinflammation contribute to immune dysfunction. Therefore, TLR4 is obviously the most suitable indicator of postfracture immune dysfunction. Furthermore, HLA-DR expression is deficient due to DNA hypermethylation in human monocytic U937 cells [31], which we chose as the cell to be studied in the model. As for the TLR4-mediated immune response, a cross-talk between HLA-DR and TLR4 has been reported [32]. Therefore, U937 cell may provide a naturally HLA-DR-deficient model to more specifically evaluate the effect of bone components on monocytic TLR4 surface expression. As shown in our result, bone components downregulated TLR4 expression as the concentration increased to 50 ng/mL. Both fracture-induced vessel tears and/or bone marrow leakage contribute to the fracture site hematoma, where monocytes, either from peripheral blood or bone marrow, may expose themselves to the bone components. Our results implicated that the nearer the bone components (thus the higher exposed concentration), the higher the possibility of TLR4 downregulation on the surface of monocytes. In patients with major trauma (injury severity score of 25 or more), expression of TLR4 on the surface of monocytes is more profoundly decreased (about 40% of healthy control) [9] than shown in our result. The clinical implications are twofold. First, our result demonstrated bone component exposure alone altered TLR4 expression in the same direction as clinical setting (downregulation). Second, the extent of TLR4 downregulation might be accentuated by other factors in addition to bone component exposure because an additive or synergistic effect on immune-suppression has been proven among fracture itself, soft tissue injury, and hemorrhage [10, 11].

The timing of the investigation for posttraumatic monocyte dysfunction is a critical issue. Although, in a rabbit model, a considerable rate of apoptosis of blood monocytes and lymphocytes occurs as early as 2 h after femur fracture, apoptotic cells disappear from systemic circulation within 24 h [33]. This means that early apoptosis of monocytes might be responsible only for initiation but not for the maintenance of postfracture immunoparalysis. In the clinical scenario, the differential blood count of patients shows unchanged relative frequencies of monocytes at 6–72 h after trauma [34]. The functional state, rather than the total number of monocytes, is responsible for traumatic paralysis of the mononuclear phagocyte system and T cell commitment [35]. Immunosuppression usually occurs at 24–48 h after trauma [20, 36]. The capacity of circulating monocytes to produce intracellular cytokines* de novo* reaches a nadir at 24 h following trauma [34]. This probably addresses the precise timing of the occurrence of immunoparalysis. Functional disturbance of circulating monocytes is alleviated at 72 h following trauma [34] due to influx of newly derived monocytes [34, 37]. Furthermore, monocyte function within 24 h of hospital admission has been incorporated into formulating the outcome predictive score [38]. Therefore, “24 h” seemed to be an appropriate time point for the study of functional disturbances of the “traumatized” monocyte. Why the LPS reactivity of monocytes reaches a nadir at 24 h after trauma remains unknown [34]. None of the following immunosuppressors, including IL-10, transforming growth factor-*β*, *β*2-agonist, catecholamine and prostaglandin E2, could fully explain the specific impairment in certain monocyte responses to LPS [39]. Most studies failed to find a correlation between plasma cytokine levels and the development of organ dysfunction because of short half-lives of cytokines or their assessment in mixed blood cell cultures. Although intracellular cytokine analysis can overcome these biological drawbacks, it has not found great clinical acceptance over the years owing to its complicated work-flow [34]. Besides, impaired LPS signaling through the CD14 coreceptor cannot account for monocyte hyporesponsiveness to LPS after trauma. Altered TLR status has been proposed as an important contributor to the decreased cytokine responses observed in trauma patients [35]. Our results validated that bone component exposure for 24 h downregulated TLR4 protein expression on the surface of monocyte in a gp96-related manner. TLR4 cycles between the Golgi and the plasma membrane of human monocytes in the resting state [22]. An inverse relationship between the cytoplasmic and membranous portions of TLR4 shown by our data suggested that the bone component caused TLR4 to accumulate during transportation and fail to express normally on the cell surface. Our results demonstrated that the expression of gp96 but not PRAT4A is reduced by bone component exposure, suggesting that TLR4-MD-2 complex formation [40] rather than TLR4 glycosylation [41] might be impaired during the insult. In addition to TLR4, gp96 can also chaperone several other TLRs, including TLR2, 5, 7, and 9 [42], on which the effect of bone component exposure has not yet been examined to evidence cross-tolerance induced by bone component exposure.

In multiple trauma patients, microorganisms can enter the circulation via alterations in the gastrointestinal mucosal barrier. Of all cases of sepsis, approximately 60% are caused by gram-negative bacteria, suggesting a critical role of bacterial LPS, endotoxin, in traumatic insults [43].* Pseudomonas aeruginosa* has been reported to be the translocating species accompanying early apoptosis of monocytes after multiple trauma [33]. Endotoxin tolerance can be induced by a wide variety of stimuli, in addition to endotoxin. Injuries, particularly multiple trauma, may also induce a state of endotoxin tolerance [44], which correlates well with trauma severity according to the monocytic response to endotoxin [43]. Our result evidenced that stimulation exclusively by the bone component itself suffices to upregulate the expression of miR-146a, a critical indicator of endotoxin tolerance [24], and the importance of bone component stimulation for the development of endotoxin tolerance in case of multiple trauma. Although endotoxin tolerance was initially thought to be a beneficial adaptive negative feedback, it may be a component of immune dysfunction complicating sepsis management. Moreover, high levels of endotoxin tolerance render them susceptible to further lethal infection [45]. It is interesting that posttraumatic immunosuppression and endotoxin tolerance share something in common. The concept of selective reprogramming rather than generalized functional suppression could be applied both in endotoxin tolerance [44] and in posttraumatic immunosuppression [9]. Both endotoxin tolerance [46] and posttraumatic immune responses [47, 48] have been shown to be independent of the TLR4 coreceptor CD14, although the TLR4 signaling pathway is altered [9, 44]. A recent review concluded that miR-146a is a negative regulator of TLR4 signaling* in vivo* and miR-146a expression parallels the level of endotoxin tolerance by regulating TLR4 downstream effectors [45]. Although there is a possibility of interaction between miR-146a and the TLR4 3′ untranslated region through cross-matching in multiple databases, our results show that bone components did not alter TLR4 mRNA stability, thus excluding the possibility of a direct action by miR-146a on the posttranscriptional modification of TLR4 mRNA expression.

Several limitations exist. Bone components were harvested from mice, not humans. Although monocytes treated with samples from human long-bone fracture sites best simulate human conditions, our model was developed according to the recently validated pseudofracture animal model [18–20], which facilitates laboratory research and animal model translation at the cellular level. Bone component solutions provided in our model were not separated into 3 parts (bone marrow cells, bone marrow supernatant, and bone suspension), because each part has been shown to cause a significant systemic inflammatory response after injection into rodent thighs [19]. In clinical situations, all 3 parts would be exposed to the injured site. The pseudofracture animal model was also developed based on the mixed bone component solution, instead of the individual component, to more adequately mimic the true fracture condition [20]. Current knowledge about the mixed bone component solution did not provide enough evidence for us to find out the key factor. Despite subdividing the mixed bone component solution into individual bone component for the study, the authors who invented the bone component solution ended up concluding that multiple cell types and ligands account for TLR4-related immune responses because of the diverse composition of the exposed bone components [19]. They also suggested that the better way to maintain homeostasis is to target it at the level of TLR4 receptor [19]. Therefore, in the present study, we used TLR4 expression as the target to evaluate the effect of mixed bone component solution on its alteration mechanisms, including transcription, posttranscriptional modification, and protein degradation as well as protein trafficking and found that gp96 took part in the mechanism regarding bone component-interfered TLR4 trafficking.

In conclusion, our cell model provides solid evidence of monocyte dysfunction by downregulation of TLR4 surface expression and upregulation of miR-146a following bone component exposure. Several applications and clinical implications exist. Firstly, manipulation of TLR4 trafficking by chaperones has been proposed as a possible medical treatment for septic shock [23], such as gp96 in the case of long-bone fracture. To further identify the gp96-related pathway mediating bone component-induced TLR4 distribution, TLR4-MD-2 association assay should be examined in this model. Secondly,* in vivo* regulation of miR-146a expression is currently possible [49, 50] and future therapeutic strategies could be aimed at fine-tuning miR-146a expression during a high-tolerance state to avoid an overwhelming secondary infection [45]. Cause-effect relationship between TLR4 downregulation and miR-146a upregulation can be further elucidated by gene manipulation. Moreover, based on our model, direction of monocyte differentiation as well as monocyte-T cell interaction following bone component exposure could also be examined to delineate the complicated trauma cascade step by step. This cell model could be reasonably utilized to bridge the pseudofracture animal model because bone components are prepared in the same way from the same source.

## Figures and Tables

**Table 1:** 

TLR4	Forward primer: 5′-AAGCCGAAAGGTGATTGTTG-3′
	Reverse primer: 5′-CTGTCCTCCCACTCCAGGTA-3′

GAPDH	Forward primer: 5′-TGCCCCCTCTGCTGATGCC-3′
	Reverse primer: 5′-CCTCCGACGCCTGCTTCACCAC-3′

## References

[1] Mathers C. D., Loncar D. (2006). Projections of global mortality and burden of disease from 2002 to 2030. *PLoS Medicine*.

[2] Krug E. G., Sharma G. K., Lozano R. (2000). The global burden of injuries. *American Journal of Public Health*.

[3] World Health Organization (2000). *Injury: A Leading Cause of the Global Burden of Disease*.

[4] Wafaisade A., Lefering R., Bouillon B. (2011). Epidemiology and risk factors of sepsis after multiple trauma: an analysis of 29,829 patients from the Trauma Registry of the German Society for Trauma Surgery. *Critical Care Medicine*.

[5] Lagoa C. E., Bartels J., Baratt A. (2006). The role of initial trauma in the host's response to injury and hemorrhage: insights from a correlation of mathematical simulations and hepatic transcriptomic analysis. *Shock*.

[6] Seely A. J. E., Christou N. V. (2000). Multiple organ dysfunction syndrome: exploring the paradigm of complex nonlinear systems. *Critical Care Medicine*.

[7] Faist E., Baue A. E., Dittmer H., Heberer G. (1983). Multiple organ failure in polytrauma patients. *The Journal of Trauma*.

[8] Baker C. C., Oppenheimer L., Stephens B., Lewis F. R., Trunkey D. D. (1980). Epidemiology of trauma deaths. *The American Journal of Surgery*.

[9] Adib-Conquy M., Moine P., Asehnoune K. (2003). Toll-like receptor-mediated tumor necrosis factor and interleukin-10 production differ during systemic inflammation. *American Journal of Respiratory and Critical Care Medicine*.

[10] Wichmann M. W., Zellweger R., DeMaso C. M., Ayala A., Williams C., Chaudry I. H. (1996). Immune function is more compromised after closed bone fracture and hemorrhagic shock than hemorrhage alone. *Archives of Surgery*.

[11] Wichmann M. W., Ayala A., Chaudry I. H. (1998). Severe depression of host immune functions following closed-bone fracture, soft-tissue trauma, and hemorrhagic shock. *Critical Care Medicine*.

[12] Hauser C. J., Zhou X., Joshi P. (1997). The immune microenvironment of human fracture/soft-tissue hematomas and its relationship to systemic immunity. *The Journal of Trauma—Injury, Infection and Critical Care*.

[13] Perl M., Gebhard F., Knöferl M. W. (2003). The pattern of preformed cytokines in tissues frequently affected by blunt trauma. *Shock*.

[14] Lazarou S. A., Barbul A., Wasserkrug H. L., Efron G. (1989). The wound is a possible source of posttraumatic immunosuppression. *Archives of Surgery*.

[15] Stahel P. F., Smith W. R., Moore E. E. (2007). Role of biological modifiers regulating the immune response after trauma. *Injury*.

[16] Mollen K. P., Anand R. J., Tsung A., Prince J. M., Levy R. M., Billiar T. R. (2006). Emerging paradigm: toll-like receptor 4—sentinel for the detection of tissue damage. *Shock*.

[17] Shalhub S., Junker C. E., Imahara S. D., Mindrinos M. N., Dissanaike S., O'Keefe G. E. (2009). Variation in the TLR4 gene influences the risk of organ failure and shock posttrauma: a cohort study. *The Journal of Trauma—Injury, Infection, and Critical Care*.

[18] Darwiche S. S., Kobbe P., Pfeifer R., Kohut L., Pape H.-C., Billiar T. (2011). Pseudofracture: an acute peripheral tissue trauma model. *Journal of Visualized Experiments*.

[19] Kobbe P., Kaczorowski D. J., Vodovotz Y. (2008). Local exposure of bone components to injured soft tissue induces Toll-like receptor 4-dependent systemic inflammation with acute lung injury. *Shock*.

[20] Menzel C. L., Pfeifer R., Darwiche S. S. (2011). Models of lower extremity damage in mice: time course of organ damage and immune response. *The Journal of Surgical Research*.

[21] Smith K. R., Klei L. R., Barchowsky A. (2001). Arsenite stimulates plasma membrane NADPH oxidase in vascular endothelial cells. *American Journal of Physiology—Lung Cellular and Molecular Physiology*.

[22] McGettrick A. F., O'Neill L. A. (2010). Localisation and trafficking of Toll-like receptors: an important mode of regulation. *Current Opinion in Immunology*.

[23] Saitoh S.-I. (2009). Chaperones and transport proteins regulate TLR4 trafficking and activation. *Immunobiology*.

[24] Nahid M. A., Pauley K. M., Satoh M., Chan E. K. L. (2009). miR-146a is critical for endotoxin-induced tolerance: implication in innate immunity. *The Journal of Biological Chemistry*.

[25] Bone R. C. (1996). Sir Isaac Newton, sepsis, SIRS, and CARS. *Critical Care Medicine*.

[26] Adib-Conquy M., Cavaillon J.-M. (2009). Compensatory anti-inflammatory response syndrome. *Thrombosis and Haemostasis*.

[27] Laudanski K., Miller-Graziano C., Xiao W. (2006). Cell-specific expression and pathway analyses reveal alterations in trauma-related human T cell and monocyte pathways. *Proceedings of the National Academy of Sciences of the United States of America*.

[28] Tschoeke S. K., Ertel W. (2007). Immunoparalysis after multiple trauma. *Injury*.

[29] Guth A. A., Hofstetter S. R., Pachter H. L. (1996). Human immunodeficiency virus and the trauma patient: factors influencing postoperative infectious complications. *The Journal of Trauma—Injury, Infection and Critical Care*.

[30] Ploder M., Pelinka L., Schmuckenschlager C. (2006). Lipopolysaccharide-induced tumor necrosis factor *α* production and not monocyte human leukocyte antigen-DR expression is correlated with survival in septic trauma patients. *Shock*.

[31] Peterlin B. M., Gonwa T. A., Stobo J. D. (1984). Expression of HLA-DR by a human monocyte cell line is under transcriptional control. *The Journal of Molecular and Cellular Immunology*.

[32] Liu X., Zhan Z., Li D. (2011). Intracellular MHC class II molecules promote TLR-triggered innate immune responses by maintaining activation of the kinase Btk. *Nature Immunology*.

[33] Efstathopoulos N., Tsaganos T., Giamarellos-Bourboulis E. J. (2006). Early apoptosis of monocytes contributes to the pathogenesis of systemic inflammatory response and of bacterial translocation in an experimental model of multiple trauma. *Clinical and Experimental Immunology*.

[34] Kirchhoff C., Biberthaler P., Mutschler W. E., Faist E., Jochum M., Zedler S. (2009). Early down-regulation of the pro-inflammatory potential of monocytes is correlated to organ dysfunction in patients after severe multiple injury: a cohort study. *Critical Care*.

[35] Spolarics Z., Siddiqi M., Siegel J. H. (2003). Depressed interleukin-12-producing activity by monocytes correlates with adverse clinical course and a shift toward Th2-type lymphocyte pattern in severely injured male trauma patients. *Critical Care Medicine*.

[36] Cohen M. J., Brohi K., Calfee C. S. (2009). Early release of high mobility group box nuclear protein 1 after severe trauma in humans: role of injury severity and tissue hypoperfusion. *Critical Care*.

[37] Johnston R. B. (1988). Current concepts: immunology. Monocytes and macrophages. *The New England Journal of Medicine*.

[38] Cheadle W. G., Mercer-Jones M., Heinzelmann M., Polk H. C. (1996). Sepsis and septic complications in the surgical patient: Who is at risk?. *Shock*.

[39] Wang J. E., Dahle M. K., Aasen A. O. (2006). Impaired monocyte Toll-like receptor-4 signaling in trauma and sepsis: is SIGIRR the answer?. *Critical Care Medicine*.

[40] Randow F., Seed B. (2001). Endoplasmic reticulum chaperone gp96 is required for innate immunity but not cell viability. *Nature Cell Biology*.

[41] Takahashi K., Shibata T., Akashi-Takamura S. (2007). A protein associated with Toll-like receptor (TLR) 4 (PRAT4A) is required for TLR-dependent immune responses. *The Journal of Experimental Medicine*.

[42] Yang Y., Liu B., Dai J. (2007). Heat shock protein gp96 is a master chaperone for toll-like receptors and is important in the innate function of macrophages. *Immunity*.

[43] Wutzler S., Maier M., Lehnert M. (2009). Suppression and recovery of LPS-stimulated monocyte activity after trauma is correlated with increasing injury severity: a prospective clinical study. *The Journal of Trauma—Injury, Infection, and Critical Care*.

[44] West M. A., Heagy W. (2002). Endotoxin tolerance: a review. *Critical Care Medicine*.

[45] Quinn E. M., Wang J. H., Redmond H. P. (2012). The emerging role of microRNA in regulation of endotoxin tolerance. *Journal of Leukocyte Biology*.

[46] Heagy W., Hansen C., Nieman K., West M. A. (2003). Evidence for a CD14- and serum-independent pathway in the induction of endotoxin-tolerance in human monocytes and THP-1 monocytic cells. *Shock*.

[47] Prince J. M., Levy R. M., Yang R. (2006). Toll-Like receptor-4 signaling mediates hepatic injury and systemic inflammation in hemorrhagic shock. *Journal of the American College of Surgeons*.

[48] Levy R. M., Prince J. M., Yang R. (2006). Systemic inflammation and remote organ damage following bilateral femur fracture requires Toll-like receptor 4. *American Journal of Physiology-Regulatory Integrative and Comparative Physiology*.

[49] Krützfeldt J., Rajewsky N., Braich R. (2005). Silencing of microRNAs in vivo with ‘antagomirs’. *Nature*.

[50] Schmelzer C., Kitano M., Rimbach G. (2009). Effects of ubiquinol-10 on microRNA-146a expression in vitro and in vivo. *Mediators of Inflammation*.

